# The Early Detection of Osteoporosis Through the Measurement of Hard Palate Thickness (HPT) Using Dental Cone Beam Computed Tomography (CBCT): A New Indicator for Osteoporosis?

**DOI:** 10.3390/diagnostics15131603

**Published:** 2025-06-25

**Authors:** Margrit-Ann Geibel, Dritan Turhani, Tilmann Blasenbrey, Meinrad Beer, Daniela Kildal

**Affiliations:** 1 Dento-Maxillofacial Radiology, Department of Maxillofacial Surgery, University Hospital Ulm, 89070 Ulm, Germany; 2 Center for Oral and Maxillofacial Surgery, Department of Dentistry, Danube University, 3500 Krems an der Donau, Austria; 3 Department of Diagnostic and Interventional Radiology, University Hospital Ulm, 89070 Ulm, Germany; meinrad.beer@uniklinik-ulm.de (M.B.); daniela.kildal@hopitalvs.ch (D.K.); 4 Radiology, Upper Valais Hospital Center (SZO), Hôpital du Valais, 3900 Brig, Switzerland

**Keywords:** dental cone beam computed tomography (CBCT), digital volume tomography (DVT), osteoporosis, hard palate thickness (HPT), palatum durum, screening method

## Abstract

**Background/Objectives**: Osteoporosis is a widespread and chronic systemic bone disease that affects the jaws and teeth and, therefore, also dentistry. Osteoporosis can be diagnosed by different radiological methods. Dental cone beam computed tomography (CBCT) plays an important role in dentistry imaging. The aim of our retrospective pilot study was to find criteria in CBCT that point to the possible existence of osteoporosis. **Methods**: Pilot study. The hard palate thickness (HPT) of the patients was measured at a defined location in the CBCT. Additionally, the CBCT images were presented to a radiologist for visual assessment. Both results were compared with the DXA measurements—as the “gold standard”—and patient history. **Results**: We found a consistent correlation between the visual assessments using established radiological criteria, including the new criterion of hard palate thickness (HPT), and the diagnosis of normal or pathological bone density. Secondly, for the HPT measurement all “pathologic” CBCT had an HPT of ≤0.9 mm, and all normal patients had an HPT of ≥0.9 mm. **Conclusions**: Despite the small sample size, this CBCT pilot study showed a correlation between HPT and systemic bone disease. Therefore, as our main result, we found a new CBCT diagnostic criterion, which quickly and uncomplicatedly points to the possible existence of bone disease, especially osteoporosis. We propose HPT as a new criterion in the evaluation of CBCT images. A threshold of <0.9 mm may be indicative for osteoporosis or osteopenia, indicating a need for further evaluation.

## 1. Introduction

The widespread disease, osteoporosis, is a chronic and systemic skeletal and bone disease, caused by a steadily increasing degradation and loss of bone tissue. Osteoporosis often goes unnoticed until the condition leads to injury.

Maximal bone density is reached between the ages of 20 and 30 [1]. With increasing age, bone density decreases and the bone structure becomes porous. This can lead to fractures as a result of even minor strains.

Osteoporosis occurs more frequently in women than in men. Postmenopausal osteoporosis, which is caused by estrogen deficiency, predominates in women, while secondary osteoporosis occurs more frequently in men. The prevalence of osteoporosis is approximately 7% in men over 50 years of age, while it is approximately 15% in postmenopausal women over 50, rising to 45% in women over 70 years of age.

The so-called “major osteoporotic fractures”, i.e., fractures that are typical of osteoporosis, increase significantly after the age of 50 in women and after the age of 60 in men [2,3].

However, data on the prevalence and incidence of osteoporosis vary considerably in the literature.

A distinction is made between primary and secondary osteoporosis.

Primary osteoporosis predominates, accounting for approximately 60% of cases. It results from a combination of risk factors, such as genetic factors; age; nutrient and vitamin deficiencies (e.g., calcium and vitamin D); increased osteoclast activity or decreased osteoblast activity; increased inflammatory factors; and, above all, a rapid decline in estrogen levels during menopause.

Primary osteoporosis includes three types: postmenopausal (Type 1); senile (Type 2); and idiopathic juvenile osteoporosis (Type 3), which is very rare and affects children and adolescents (between eight and thirteen years of age).

The secondary form of osteoporosis is the result of another disease or its treatment.

Typical diseases include the following: diabetes mellitus, hypogonadism, hyperthyroidism, Cushing’s syndrome, hyperparathyroidism, chronic inflammation or kidney disease, multiple sclerosis, Parkinson’s disease, malabsorption syndromes or malnutrition, cancer, and congenital connective tissue diseases (e.g., Marfan syndrome and osteogenesis imperfecta). Medications that promote secondary osteoporosis include the following: glucocorticoids, SSRIs, antiepileptics, proton pump inhibitors, and aromatase inhibitors.

A suspected diagnosis of osteoporosis is made based on medical history (e.g., risk factors such as age, gender, and previous fractures); clinical findings; laboratory findings (e.g., previous fractures with reduced height or postural changes); and diagnostic tests.

If fractures have already occurred due to reduced bone density, this is referred to as manifest osteoporosis. Although X-rays can be very helpful, they are unable to reliably diagnose or quantify osteoporosis. X-rays and CT scans are particularly insensitive in the early stages, and their use in differentiation from osteopenia is also not possible in later stages. The gold standard for osteoporosis diagnosis is, therefore, bone density measurement.

### 1.1. DXA

The condition is definitively diagnosed by measuring the bone mineral density of the lumbar spine, hip, and/or radius using quantitative CT or dual-energy X-ray absorptiometry (DXA), which is considered the standard method for osteoporosis diagnosis [4,5,6]. The unit for bone mineral density (BMD) in DXA is the areal density (g/cm^2^). For diagnostic classification, the BMD is converted into a T-score and a Z-score. These scores are used in the diagnosis of osteoporosis and osteopenia, using BMD reference data [5]. The T-score describes the patient’s BMD as the standard deviation (SD) from the mean value in young (20–29 years old), healthy women [6]. The Z-score corresponds to an age comparison, i.e., the patient’s BMD compared to a reference group of the same age. The diagnosis is determined by the lowest T-score in one of the recommended DXA regions [5]. T-scores of ≥−1.0 SD are considered normal BMD. Osteopenia is present at a T-score of between −2.5 SD and −1.0 SD in the young reference population. The WHO has defined a threshold value of 2.5 SD below the mean BMD of young adult women as a criterion for osteoporosis [5,6].

### 1.2. T-Score Classification

Diagnosed osteoporosis, according to the WHO, can be divided into grades 0–3. In grade 0 osteopenia, the T-score is between −1 and −1.5, and no fractures are known. In grades 1–3, the T-score is below −2.5. In grade 1, preclinical osteoporosis, no fractures are known. In grade 2, known as manifest osteoporosis, there are between one and three vertebral fractures. In grade 3, advanced osteoporosis, there are multiple vertebral and extremity fractures [7].

### 1.3. QCT

Clinical whole-body CT scanners are used for QCT of the spine and hip.

Bone density measurements using QCT are performed by measuring Hounsfield units at L1–L3, provided these are not already fractured. The conversion to absolute density is performed using a reference phantom. The results are expressed in mg/mL. The following threshold values apply: osteopenia is defined as <120 mg/mL and osteoporosis is defined as <80 mg/mL [8]. Because QCT measures trabecular bone density, which changes significantly in osteoporosis, this measurement method is more sensitive than DXA, but less readily available. The radiation exposure from taking QCT images of hips is also significantly higher, at 2.5–3 mSv [9], than from taking DXA measurements [10]. QCT can be used to predict fracture risk and monitor bone density during therapy, but current guidelines [7] recommend using DXA.

### 1.4. Osteoporosis in Dentistry

For dentists, osteoporosis is also a relevant disease, which can be reliably identified. Metabolic osteopathologies, such as osteoporosis, affects the entire skeleton, and, thus, also the jaw and teeth. Therefore, cavities and the loss of teeth accumulate among patients with osteoporosis [11,12]. Dental research shows that osteoporosis can affect the teeth in noticeable ways, including tooth loss and gum disease.

Osteoporosis is also a known and very important factor in regard to the success of dental implants. Healthy bone metabolism is the prerequisite for the osteointegration and long-term maintenance of dental implants, in order to reduce the risk of a rejection reaction and, thus, the loss of the implant. Local bone density is an important factor in the primary stability of the implant [13]. This indicates that osteoporosis may be a risk factor for osseointegration [14]. For patients with implants, dentures, and bridges, weak bones may lead to looser-fitting replacements and also to longer healing times [15].

The presence of osteoporosis is also an important factor in periodontal therapy [16,17]. Before periodontal therapy is initiated, all remaining teeth must be examined for degrees of loosening to ensure its success. These degrees are divided into loosening Grade 1 for “slight loosening”, loosening Grade 2 (moderate loosening, but worth preserving), and loosening Grade 3 for “severe loosening”. Thus, a finding of Grade 3 in a tooth does not indicate a need for preservation [16,17]. If osteoporotic disease is present, not only should the Grade 3 teeth be extracted before periodontal therapy, but the remaining teeth with Grade 2 findings should also be carefully evaluated for preservation [16,17].

Unfortunately, certain medications for osteoporosis, such as bisphosphonate drugs, can cause dental issues—something all doctors should be aware of when prescribing any medications [18].

Diagnostic correlations between osteoporosis and specific changes in the jawbone were already described by White et al. in 2002 [19], but the sensitivity and specificity of the methods described do not seem sufficient for clinical application [19,20]. Later studies used two-dimensional dental X-ray images [21] and dental cone beam computed tomography (CBCT) to identify patients at risk of osteoporosis [22]. CBCT is suitable for the precise visualization of a wide range of dental diseases and improves diagnostic accuracy in periodontics, orthodontics, endodontics, and implantology. CBCT thus leads to improved treatment planning and, ultimately, to better patient outcomes [23]. CBCT is, therefore, widely used and applicable today.

Actual dental methods for the detection of osteoporosis include the following:

- Comparisons between the bone density between the roots of the teeth on intraoral dental films and a defined metal reference object [24,25,26].
- Measurements of the height of the corticalis in the mandibula and underneath the mental foramen in a panorama tomography, or comparisons of the mandibular cortical shape according to Klemetti [8].
- Comparisons of the morphology of the compact lower mandible in CBCT (CT cortical index); calculations (CT mandible indexes) of Koh and Kim (2011) [22]; or the panoramic mandibular index (PMI) of Benson et al. (1991), which can disclose osteoporosis [27].
- Measurements of the Hounsfield units (HU) in the spongiosa distal of the mental foramen (cone beam CT density index) [28].

In some calculations, age is also included [24]. These methods appear suitable for excluding osteopenia or osteoporosis [29], but they cannot reliably diagnose or differentiate osteopenia or osteoporosis [30].

The measurements and methods described here have the disadvantage in daily practice of needing very precise and sometimes multistage measurements, which require too much work and time; therefore, they are unsuitable for a quick check for osteoporosis in the framework of incidental findings. Thus, there is a need to develop a fast method of screening to make the appraisal of osteoporosis feasible for dentists in daily practice [31].

Further problems arise from the diversity of methods; to our knowledge, there is currently no universally accepted standard for the diagnosis of osteoporosis in dental imaging. Osteoporosis diagnosis through dental examinations should be simple, rapid, and inexpensive in order to achieve the highest possible acceptance.

### 1.5. New Methods

Refs. [32,33]: AI-based systems for diagnosing osteoporosis using dental imaging are increasingly being tested, as two meta-analyses show. Modular neural networks, as proposed by Namatevs et al. (2023), have been shown as promising in detecting osteoporosis in mandibular CBCT scans, suggesting the potential for future integration in diagnostic tools [34].

Although the accuracy of AI in osteoporosis diagnosis is sometimes high, the results are not yet sufficiently reliable and exhibit very high variability in sensitivity and specificity. The widespread application of AI-based systems in osteoporosis diagnosis is currently not available.

Before the development of DXA, osteoporosis was diagnosed using X-rays. For example, the trabecular patterns of the clavicula, femur, hip, calcaneus, or combinations of these were analyzed. It has also long been known that osteoporosis leads to cortical thinning and a reduced cancellous bone-to-cortical ratio of the metacarpal bones and long bones [35,36,37,38].

More recent studies [39] have shown a correlation between the cortical index used to calculate the cortical thickness and bone mineral density (BMD) measured by DXA in the tibia, metacarpal bones, and mandible.

These methods mainly refer to long bones; flat bones were not considered. A computed tomography (CT) study from 2024 showed a correlation between the hard palate (flat bone) thickness and diseases associated with a general change in bone density. In osteopetrosis (a congenital ossification disorder that leads to a massive increase in bone density due to the disruption of osteoclast activity, with reduced bone resorption while bone formation is preserved) HPT was increased. In osteopenia and osteoporosis, it was decreased in comparison to a normal collective, consisting of young, healthy subjects [40]. We did not find any further literature about changes in the palatum durum due to osteoporosis, but it can be assumed that the maxilla and palatine bone in case of osteoporosis show similar structural and morphological changes as other bony structures, such as spongiosa reduction and decreased cortical thickness.

For routine dental imaging, CBCT is the safer and more efficient option compared to CT, with lower radiation exposure and high-quality diagnostic images [41,42]. In addition, the resolution of a CBCT image with a slice thickness of up to 0.2 mm is higher than examination using magnetic resonance imaging (MRI) or computed tomography (CT) with 0.5 to 3 mm. As a result, measurement errors in small volumes should also be smaller compared to CT.

### 1.6. Purpose

The aim of our pilot study was to find diagnostic criteria for dentists in CBCT, which quickly and uncomplicatedly point to the possible existence of osteoporosis, ideally with the reuse of existing treatments. This may help to identify osteoporosis at an early stage, resulting in early referrals for corresponding clarification.

We chose the hard palate as the object to investigate as an anatomical structure because it is clearly definable, simple to find, and appears in many CBCTs and CTs. The measurement of the thickness (HPT) is quite simple and was performed in our study via computer-supported measurement on one defined measurement location in the Processus palatinus maxillae.

## 2. Methods

This retrospective pilot study was based on an RIS/PACS query. The patients were identified by an RIS-based, full-text search for CBCT images. In addition, the patient histories were checked for bone diseases and DXA diagnoses.

The Ethics Committee reviewed and approved our study (357/12).

We defined a patient group with CBCT and DXA measurements, as well as a control group of young (<30 years old) patients with CBCT images but no known systemic skeletal diseases, particularly without evidence of osteoporosis. The control group was formed of 52 young patients with healthy bones, according to the WHO definition of osteoporosis. They, thus, represented the normal state—the T-score regarding bone density. Due to the small number of patients with CBCT and DXA images, we were not able to find a sufficiently large cohort of healthy older patients for this pilot study, which would have allowed for a comparison with patients of the same age and in terms of the Z-score.

For the patient group, 126 dental images/patients with CBCT imaging could be found. All CBCT images that were found were used, regardless of the CBCT scanner used or the original indication for the examination. The voxel sizes and FOV varied accordingly. All examinations were evaluated in three planes, with measurements taken at maximum possible magnification in sagittal views.

For the visual assessments, 52 out of 126 patients had to be excluded because of the following:

- Their hard palate was not recorded in their CBCT images;
- They had bone deformities, due, for example, to fractures, tumors, torus palatinus, or cleft palates;
- There were impediments to measurement, such as chronic or acute inflammatory diseases of the os palatum or maxillary sinuses;
- There were motion artifacts or overlying metal artifacts from dental prosthetic materials, braces, or temporary anchorage devices.

For the HPT measurement, another 6 patients had to be excluded because the HPT measurement point was not captured. In total, 74 (visual assessment) and 68 patients (measurement) could be analyzed, as follows:

- 52/52 control group (young patients < 30), with normal bone mineralization and no known history of bone disease;
- 18/12 patients with osteoporosis/osteopenia (pathologic), based on DXA;
- 4/4 patients > 30 years old with normal bone mineralization.

All CBCT images/patients were presented independently, without any additional information and in a controlled, blinded way, once to a radiologist and once to a dentist for the following reasons:

(1) To measure the HPT,
(2) For the visual assessment of a possible bone disease.

**Figure 1:**
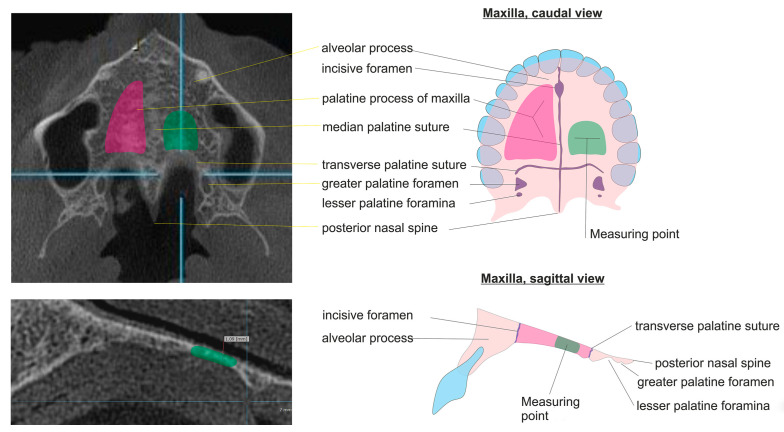
Measurement point for HPT on the left dorsal third of the palatal process of the maxilla.

The HPT was measured at maximum magnification in the Picture Archiving and Communication System (=PACS). We defined the measurement point for HPT on the left dorsal third of the palatal process of the maxilla because the contour of the hard palate is linear here over approximately 1 cm (see Figure 1).

The applied criteria for the visual assessments of possible bone disease were as follows:

- Increased radiation transparency of the imaged section of bones;
- Rarefication of the depiction of the spongiosa in the jaw;
- Decreasing thickness of the corticalis in the jaw;
- Formation of the lengthwise trabeculae in vertebral bodies, here mostly the dens;
- Reduction in the thickness of the hard palate (new criterion).

**Figure 2:**
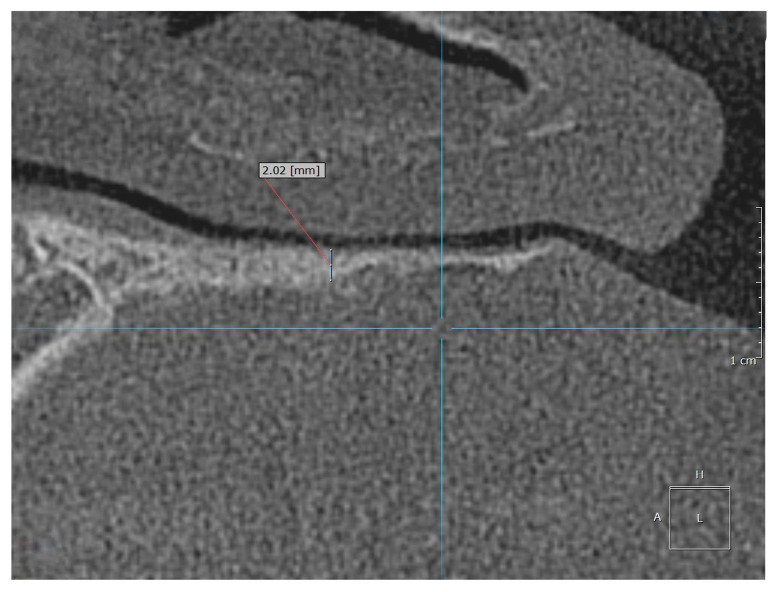
Example of the measurement location for a normal patient.

**Figure 3:**
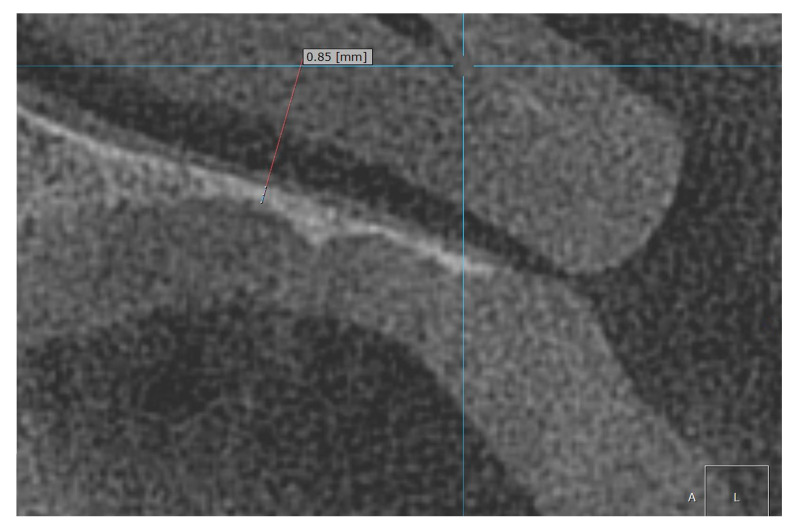
Example of the measurement location for an osteopenic patient.

**Figure 4:**
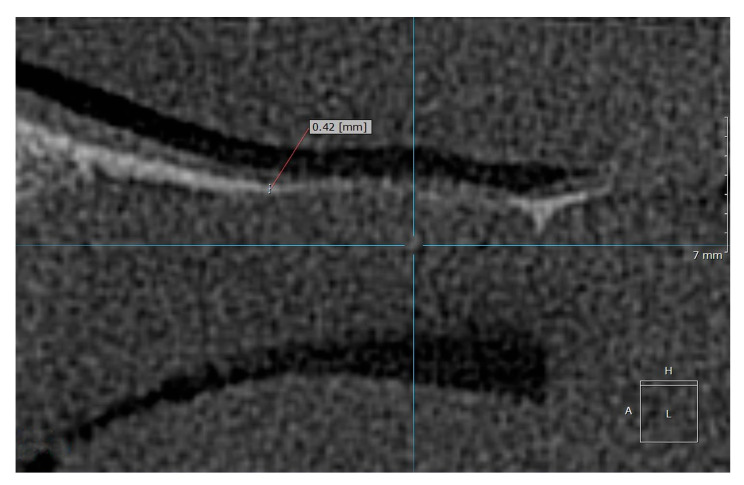
Example of the measurement location for an osteoporotic patient.

Examples of the measurement of normal, osteopenic, and osteoporotic patients are shown in Figure 2, Figure 3 and Figure 4.

The HPT measurements were investigated against the diagnosis based on the bone density measurements (DXA), as the gold standard. The visual assessment results were compared against the diagnosis based on the bone density measurements (DXA) and patient history.

The control group included patients without any known history of bone disease. The young and healthy patients (age < 30) were taken as a reference for the normal patients, in the same way as the WHO defined bone density measurement.

The DXA results were used as for the diagnoses of the pathologic patients in the DXA group. According to the WHO, the T-scores represent numbers that compare the condition of an older person’s bones with those of an average young person with healthy bones (mean^WHO^). A young person here means healthy 25- to 35-year-old adults. The T-score of x is mean^WHO^ −x SD. The T-score categories were a T-score ≥ −1 indicated normal bone density; a T-score between −1 and −2.5 indicated low bone mass—osteopenia; and a T-score ≤ −2.5 SD indicated the presence of osteoporosis [5,6]. The WHO approach to T-scores was applied in an analogous way to HPT measurement. According to the WHO, the T-score definition of bone density measurement > mean^WHO^ −1 SD, indicates normal bone density. Applied to the HPT measurement results in the HPT measurement values, ≥ mean^HPT^ −1 SD indicated normal bone density. The HPT measurement values < mean^HPT^ −1 SD and > mean^HPT^ −2.5 SD, indicated low bone mass. HPT measurement values ≤ mean^HPT^ −2.5 SD indicated the presence of osteoporosis. Since this pilot study was about finding an early indicator for osteoporosis, the more important classification, HPT measurement values = mean^HPT^ −1 SD, was used as a cutoff between normal bone density and low bone mass and the risk of osteoporosis. From here, an HPT cutoff value or line was derived to distinguish between normal and suspected osteoporosis.

### Data Analysis

The statistical analysis was performed using Microsoft 365 Excel, Version 2022. Data were expressed as mean values and range (min/max), mean, SD (standard deviation), and median. Scatter diagrams were used to show dependencies and correlations. The HPT measurements and derived diagnosis results were compared against the gold standard, as defined by DXA. Therefore, sensitivity (Sens), specificity (Spec), positive predictive value (PPV), negative predictive value (NPV), and the confidence interval (CI) were used as quality indicators.

## 3. Results

### 3.1. Visual Assessment

In the visual assessment, using the established criteria of a possible bone disease and the new criterion, “optical reduction of the thickness of the hard palate”, the radiologist identified 56 (of 74) images as normal. Osteopenia was suspected in 7 (of 74) and osteoporosis in 11 (of 74) cases. Overall, 97% of the images (72 out of 74) were correctly diagnosed as normal or pathologic against the DXA diagnosis. All 52 images of the control group and the images of the four normal patients were correctly classified as normal.

In total, 11% (2 out of 18) of the pathologic images were misclassified as having normal bone mineralization, one patient with osteoporosis and one with osteopenia (according to the DXA measurement).

In total, 89% (16 out of 18) of the patients with osteopenia or osteoporosis were correctly diagnosed as “pathologic” in the visual assessment. The distinction between patients with osteopenia and osteoporosis was not reliable in the visual assessment: 3 out of the 6 patients with osteopenia (50%) were classified as having osteoporosis, and 1 out of the 10 patients with osteoporosis (10%) was classified as having osteopenia.

### 3.2. HPT Measurement

The HPT was measured in the CBCT images by a radiologist and a dentist, independently. The defined measurement point (see Figure 1) was applied for 68 CBCT images: 52 normal and young (control group), 4 normal > 30 years old patients with DXA, and 12 patients with osteopenia or osteoporosis with DXA.

In the control group and normal patients, the mean HPT (mean^HPT^) was 1.99 mm (median = 2.07 mm, SD = 0.57). In the group of patients with osteopenia or osteoporosis, the mean HPT was 0.57 mm (median = 0.6 mm, SD = 0.18). For osteopenia and osteoporosis, the subgroups could not be distinguished as the mean HPT was =0.55 mm for osteopenia and =0.58 mm for osteoporosis.

**Figure 5:**
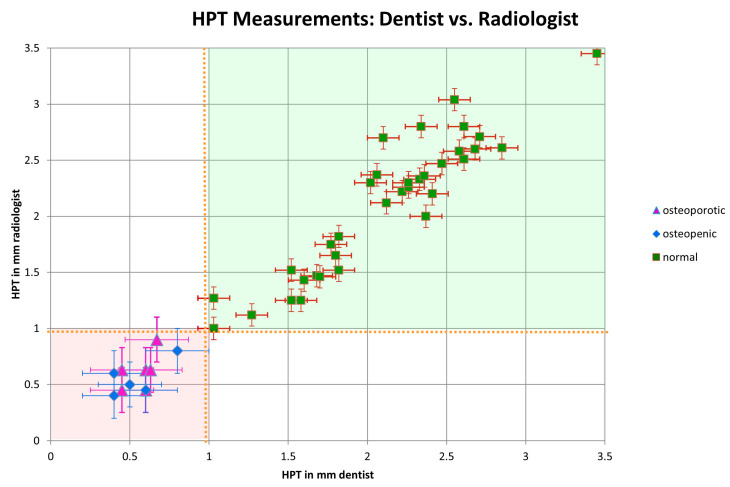
HPT measurements of the dentist in comparison to the radiologist in mm, including pathologic (light red: osteoporosis/osteopenia) and normal (light green) areas.

**Figure 6:**
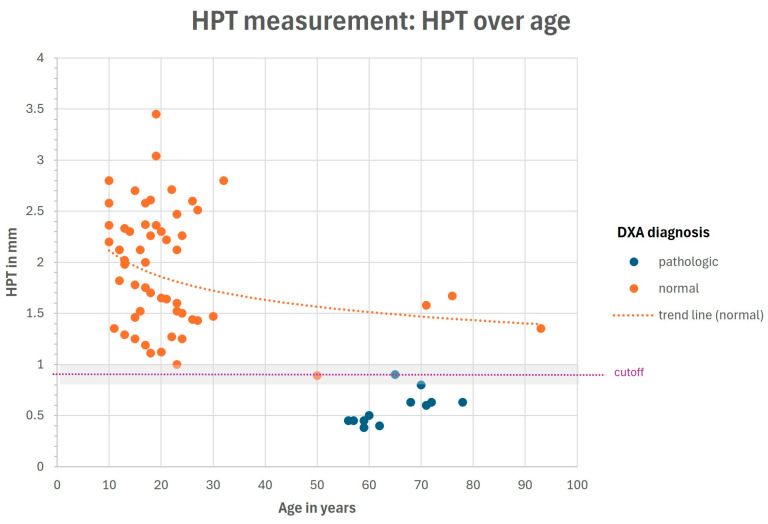
HPT measurements in mm, over age of patients in years.

The HPT measurement values in the control group and normal patients were between 0.9 and 3.5 mm, and were between 0.4 and 0.9 mm for the pathologic patients (osteopenia/osteoporosis) (see Figure 5 and Figure 6).

Applying the WHO T-score approach [5,6], mean^HPT^ −1 SD (=1.42 mm), the suggested cutoff for normal was 1.42 mm and mean^HPT^ −2.5 SD (=0.56 mm). The cutoff for osteoporosis was 0.56 mm, which led to the same diagnosis as the DXA diagnosis. Further considerations in more detail were used to compare the HPT measurements with the DXA diagnoses. Here, DXA was used as the gold standard. All patients with HPT ≥ 0.9 mm were classed as normal (Sens = 1). In this case, we would have mean^HPT^ −1.9 SD, in analogy to the WHO’s approach.

Taking a cutoff of HPT^cutoff^ = 0.84 mm, all patients with HPT ≤ 0.84 mm were considered pathologic (Sens = 1, Spec = 0.98, NPV = 0, and PPV = 0.92), corresponding to mean^HPT^ −2 SD. If the cutoff derived from HPT was −1 SD or above, this indicated normal bone density. HPT^cutoff^ −2 SD indicated low bone density or osteoporosis, which was in-line with −1 SD or −2.5 SD of the WHO’s T-score. For an HPT^cutoff^ below −2.5 SD, all patients have osteopenia or osteoporosis, according to the DXA diagnosis.

It should be noted that the distinction between the patients with osteopenia and osteoporosis was not reliable with the HPT measurement (see Figure 5 and Figure 6).

## 4. Discussion

This study’s findings were compared with previous works on the mandibular cortical index (MCI) and the panoramic mandibular index (PMI), suggesting that flat bone structures, such as the palate, can offer additional diagnostic value. Several studies have explored the potential of CBCT imaging in osteoporosis detection. The resolution of CBCT imaging allows the accurate measurement of small anatomical structures, such as the hard palate.

This was also demonstrated in CBCT studies examining soft tissue changes in craniofacial anomalies [43].

The use of CBCT to assess bone density has been evaluated in several systematic reviews. Poiana et al. (2023) concluded that CBCT is a valuable diagnostic adjunct for detecting low bone density, especially in combination with standard indices [44]. Isayev et al. (2023) provided a comprehensive systematic review supporting CBCT’s predictive value for osteoporosis in postmenopausal women [45]. In the studies compared here, the grayscale values of CBCT were compared with the results of dual-energy X-ray absorptiometry.

However, this requires sufficient bone thickness for the placement of the ROI and, of course, comprehensive knowledge of the measurement points and the standard values for density measurements. In addition, a sufficient routine for using the measurement tools is required; measuring the height of a bone with a ruler, as we described with HPT, appears easier than measuring the bone density with ROI.

Even without measurement, in the visual assessment of the CBCT images—when applying the well-known radiological image criteria and the new criterion “optical reduction in the thickness of the hard palate”—89% of the patients with osteoporosis or osteopenia were correctly diagnosed and all normal patients were correctly diagnosed, in comparison to the DXA diagnosis. This means that visual radiological diagnosis (using HPT but without measurement) is already quite reliable. However, a differentiation between osteopenia and osteoporosis is not possible. The applied criterion, “decrease in the hard palate thickness”, is not normally used, according to the literature search, with only one other study being found. The radiologist found this new criterion very helpful, especially since CT images of the jaw are rarely used to diagnose osteoporosis. The cervical vertebrae (axis/dens) visible here tended to show increased sclerosis due to degeneration. Therefore, from a purely radiological perspective, relatively few reliable structures can be found with regard to possible osteoporosis. The application of cortical indices or measurements in the typically imaged area is limited due to the lack of long bones. The methods described above primarily refer to long bones; flat bones were not considered. Yet it is comprehensible that the decreasing thickness of the corticalis of a flat bone leads to a decrease in the height [40]. The HPT, consequently, showed a dependency on normal bone density or reduced or low bone density based on a comparison with the DXA diagnosis, which allowed us to distinguish between normal and an indication of osteoporosis.

Due to the relatively small number of patients in this pilot study, it was not possible to establish a standard value distribution specific to age and/or gender. This will be reserved for larger subsequent studies.

One limitation may have been the use of PACS-based measurement software, which is unavailable in some practices, or the fact that different PACS systems (from different manufacturers/providers) are used in different clinics. However, common image viewing programs today are generally equipped with sufficiently accurate measurement tools.

Identifying the measurement site is sometimes difficult due to anatomical differences, which limits reproducibility. However, in our study, these difficulties proved to be minimal, and the measurement deviations between the two examiners were smaller the lower the HPT was. Overall, the result—pathological or non-pathological—did not appear to be influenced in most cases. Nevertheless, these problems can lead to a lack of accuracy. Since the sample size of the pilot study was small, larger studies are needed to improve the measurement method. AI-assisted measurements, with many measurement points and an average value calculated from them, should also be considered. This could result in the greater accuracy of HPT in the anatomical target range.

A differentiation between osteoporosis and osteopenia was not possible. This is comparable to other radiological methods. It is also unnecessary, as osteoporosis must be verified using DXA, as it is the gold standard. Any osteoporosis therapy would also be monitored over time using DXA, so DXA does not lose its significance. Larger studies may be able to find a better differentiation, although, for the reasons mentioned above, this would not be absolutely necessary. We have a criterion here that, in the sense of a rapid “screening”, can be considered a possible visual diagnosis, indicating the presence of a bone disease, particularly osteoporosis. It should, therefore, lead to further investigation.

The DXA T-score approach was applied in a similar way to the WHO for osteoporosis, which allows the comparison of young and healthy persons with older pathologic patients. Both groups were selected from the same pool, without additional selection criteria, which was in line with the WHO’s approach to minimizing other dependencies. Of course, the known impact factors and dependencies for osteoporosis are valid here as well; therefore, a specific verification of all the possible impacts was not part of this pilot study. All patients with a osteopenia/osteoporosis DXA diagnosis had a thickness in their hard palate (max. 0.9 mm). In contrast, all patients in the control group and all patients with normal DXA had a thickness of at least 0.9 mm. Considering a measurement fault of ±5% (±10%), and, if we draw the cutoff line at 0.9 mm, we can define an HPT grey area with false negative or false positive results as between 0.855–0.945 mm (0.81–0.99 mm). For the grey area or the error of measurement, it can be treated the same as for the normal or pathologic groups. The main reason is that absolute measurements are less accurate than relative measurements. In this sense, it might be necessary to adapt the criteria individually. Of course, the individual error of the HPT measurement can be different, mainly because of the accuracy and having to find the right measurement location. It would be good to recommend that the HPT measurement point is included in the CBCT by default—the benefit seems to outweigh the consideration of the effective doses [46]. The measurement of the defined measurement point itself also proved to be difficult, as disruptions were present from illnesses of the teeth or maxillary sinuses in a considerable number of patients. A further difficulty was that the hard palate often was not imaged in the CBCT for reasons of radiation protection, for example, in the CBCT before dental implants in the lower jaw. Should further studies show that the HPT is suitable for the diagnosis of osteoporosis, an alteration in the procedure would be advisable for patients at risk of this disease.

## 5. Conclusions

In summary, and despite the small sample size, the results of this pilot study were very interesting. HPT is not yet a well-known criterion, but it seems to be helpful and should be examined in further studies. Radiologists can clearly recognize pathological bone density when applying radiological criteria and the thickness of the hard palate. The measurement of HPT could be further improved through additional studies. The differentiation between osteopenia and osteoporosis was, as with other radiological methods, not certain in this study. Therefore, the final diagnosis should be confirmed by a measurement of the bone density. Larger studies could potentially find a precise definition of thresholds, although we believe this is not absolutely necessary. HPT is a criterion that can be used for rapid screening—a quick, simple measurement, which can indicate the presence of bone disease, particularly osteoporosis, prompting further investigation. The exact classification of osteoporosis and therapy monitoring are based on DXA values, so DXA does not lose its importance.

In conclusion, this method offers a rapid and economic approach for osteoporosis screening. However, it requires further validation through a larger, more diverse sample and clinically available tools. The use of AI also seems promising in making the measurement results even more accurate.

Patients that may be affected by osteoporosis or osteopenia can be identified safely, simply, and easily by means of a measurement of the HPT. No further calculations or reconstructions are necessary, which makes the procedure very simple and economical.

## Data Availability

Data supporting the reported results can be found in the hospital’s PACS system.
